# Pectin and Zinc Alginate: The Right Inner/Outer Polymer Combination for Core-Shell Drug Delivery Systems

**DOI:** 10.3390/pharmaceutics12020087

**Published:** 2020-01-21

**Authors:** Giulia Auriemma, Andrea Cerciello, Rita P. Aquino, Pasquale Del Gaudio, Bruno M. Fusco, Paola Russo

**Affiliations:** Department of Pharmacy, University of Salerno, Via Giovanni Paolo II 132, I-84084 Fisciano (SA), Italy; gauriemma@unisa.it (G.A.); acerciello@unisa.it (A.C.); aquinorp@unisa.it (R.P.A.); pdelgaudio@unisa.it (P.D.G.); fuscobr@unisa.it (B.M.F.)

**Keywords:** natural polysaccharides, betamethasone, core-shell particles, inflammatory bowel disease

## Abstract

Core-shell beads loaded with betamethasone were developed using co-axial prilling as production technique and pectin plus alginate as polymeric carriers. During this study, many operative conditions were intensively investigated to find the best ones necessary to produce uniform core-shell particle systems in a reproducible way. Particularly, feed solutions’ composition, polymers mass ratios and the effect of the main process parameters on particles production, micromeritics, inner structure, drug loading and drug-release/swelling profiles in simulated biological fluids were studied. The optimized core-shell formulation F5 produced with a pectin core concentration of 4.0% *w/v* and an alginate shell concentration of 2.0% *w/v* (2:1 core:shell ratio) acted as a sustained drug delivery system. It was able to reduce the early release of the drug in the upper part of the gastro-intestinal tract for the presence of the zinc-alginate gastro-resistant outer layer and to specifically deliver it in the colon, thanks to the selectivity of amidated low methoxy pectin core for this district. Therefore, these particles may be proposed as colon targeted drug delivery systems useful for inflammatory bowel disease (IBD) therapy.

## 1. Introduction

Inflammatory bowel disease (IBD) includes almost two disorders: ulcerative colitis and Crohn’s disease [1,2]. The conventional treatment of IBD is based on the regular intake of anti-inflammatory drugs and the daily administration of high drug doses is required [3]. Corticosteroids (CS), are included in the standard treatment of IBD, thanks to their potency of action as well as low dosage requirement.

The use of conventional drug delivery systems in IBD is limited, since they lead to systemic absorption of the drug, with related side effects and toxicity [4], such as osteoporosis and osteonecrosis, adrenal suppression, diabetes, cataract and glaucoma, as well as gastro-intestinal, cutaneous and cardiovascular complications [5]. As a consequence, several innovative drug delivery systems have been developed to achieve a selective drug delivery to the inflamed tissues [6,7,8,9]. With this aim, several approaches can be followed, i.e., the use of prodrugs becoming active after the hydrolysis by specific colon enzymes [10], bioadhesive delivery systems [11], timed-dependent delivery systems [12] and drug coating with pH dependent polymers [13,14]. In this case, the use of pH-sensitive polymers protects the drug from the early release in the acidic pH of the stomach and small intestine, increasing the amount of active compound in the colon region. 

In the field of oral controlled-release dosage forms, natural polysaccharides such as alginates [15], pectins [16,17], natural gums [18], chitosan [19,20] or cellulose derivatives [21] continue to occupy a prominent role, thanks to their biocompatibility, biodegradability, good biological performance and enzyme-controlled biodegradability [22]. Moreover, they have a pH-dependent solubility [23,24] and are able to swell and form gels, offering a large number of possibilities to modulate the release of loaded active compounds, according to the different therapeutic needs [25,26,27].

The main technological approaches used to produce polysaccharides-based Drug Delivery Systems (DDS) include spray drying [28], spray-chilling [29], freeze-drying [30,31], coacervation [32], emulsification followed by evaporation or solvent extraction [33] and prilling [34,35]. Among them prilling, also known as laminar jet break-up, has been considered a proper tool in creating polymeric hydrophilic microparticles (gel-beads) as modified delivery devices [36]. This technique essentially consists of two operations: the first is based on the breaking apart of a laminar jet of polymer solution into a row of mono-sized drops by means of a vibrating nozzle device, the second on their “solidification” through the ionotropic gelation method, exploiting the ability of polyelectrolyte polymers to cross-link in presence of counter ions [37,38,39]. Prilling technology offers different advantages; it is a mild and easy scalable microencapsulation technique that, if used in the co-axial configuration and in the proper operative conditions, could be used to obtain, in a single productive step, particles with multiple layers of different polymeric materials [40].The appropriate combination of two or more polysaccharides may be an effective way to prevent the early release of the drug in the upper part of the gastro-intestinal tract (GIT) that, as well known, represents the major drawback of oral formulations based on single polysaccharides [20,41,42,43]. In addition, a multilayered system offers the possibility to release the drug in a specific district of the organism or in a precise moment of the day as required, for example, for the treatment of severe chronic mucosal inflammations such as IBD. 

In this paper, we exploited the co-axial prilling technique for the manufacturing of core-shell beads, starting from amidated low methoxy (ALM) pectin as core material and sodium alginate as coating polymer. The aim was to combine the pH dependent solubility (gastro-resistance) of zinc alginate and the colon targeted selectivity of zinc-ALM pectinate [17,44] in a unique system to obtain a successful enteric carrier. Particularly, Zn^2+^ was used as alternative cross-linker because it is able to produce stronger pectinate network as reported in some studies [45,46] and showed improved stability in the upper gastro-intestinal tract [47]. Betamethasone (B) was selected as model active pharmaceutical ingredient (API) to load within the core. It is a long-acting corticosteroid with a very high anti-inflammatory potency also at low dosage and commonly used for the treatment of inflammatory bowel disease. During this research many operative conditions were intensively investigated to find the best ones necessary to produce uniform core-shell particle systems in a reproducible way. Particularly, feed solutions’ composition, polymers mass ratios and the effect of main process parameters on particles production, micromeritics, inner structure, and drug loading were studied. Finally, in vitro drug release and swelling profiles were studied with the aim to predict in vivo drug release behaviour in the gastro-intestinal tract (GIT).

## 2. Materials and Methods

### 2.1. Materials

Sodium alginate (alg) (European Pharmacopoeia X, MW ≈ 240 KDa) was obtained from Carlo Erba (Carlo Erba, Milan, Italy); amidated low methoxy (ALM) pectin (pct) (esterification degree 24% and amidation degree 23%) was kindly offered by Herbstreith and Fox KG (Neuenburg, Germany); zinc acetate dihydrate was supplied from Sigma-Aldrich (Sigma-Aldrich, Milan, Italy). Betamethasone (B) was provided by Carbosynth (Compton, United Kingdom). All other chemicals and reagents were obtained from Sigma Aldrich and used as supplied.

### 2.2. Core and Core/Shell Beads Preparation

Drug loaded formulations were prepared by dissolving the polymers (alginate or pectin) in deionized water at room temperature under gentle stirring in order to obtain polymer solutions with alginate concentrations of 1.0%, 1.5% and 2.0% (*w/v*) and pectin concentration of 4.0% (*w/v*). After polymer hydration, B was added to the feed solution in order to obtain a drug/polymer ratio of 1:20. Betamethasone loaded beads were obtained by a Nisco Encasulator apparatus (Var D; Nisco Engineering Inc., Zurich, Switzerland), coupled to a syringe pump (Model 200 Series, Kd Scientific Inc. Boston, MA, USA). The polymeric solutions were pumped through a 600 µm nozzle for “only core” beads, produced as control, and a co-axial nozzle system for core-shell beads (400 µm inner and 600 µm outer diameter, respectively). The experiments were performed at the following volumetric flow rates: 5mL/min for “only core” formulations and 6 mL/min (core), 10 mL/min (shell) for core/shell beads. The vibration frequency used to break up the laminar liquid jet was set between 250 and 350 Hz, amplitude of vibration 100%. The distance between the vibrating nozzle and the gelling bath was fixed at 25 cm. A stroboscopic lamp was set at the same amplitude as the frequency, in order to visualize the falling droplets. These latter were gelled in an aqueous solution of zinc acetate (concentration = 10% *w/v*, pH = 1.5, cross-linking time = 8 min) under gentle stirring. Afterwards, the gelling bath was filtered to recover the obtained particles, rinsed with deionized water. Finally, beads were dried at room conditions (22 °C; 67% RH) for several hours (12–18 h) up to a constant weight. Moreover, as a control, blank beads were obtained, using polymeric solutions not containing the active compound, according to the experimental protocol described above. 

### 2.3. Beads Size and Morphology

Size distribution of dried beads was obtained by scanning electron microscopy (Carl Zeiss EVO MA 10 microscope with a secondary electron detector. Carl Zeiss SMT Ltd., Cambridge, UK and a LEICA EMSCD005 metallizator), also used to observe the morphology of the particles. Analysis was conducted at 20 KeV. Projection diameter of dried beads was obtained by image analysis (Image J software, Wayne Rasband, National Institute of Health, Bethesda, MD, USA). A minimum of one hundred beads images were taken for each batch, in order to obtain particles diameter and relative standard deviation for at least three different prilling processes. Perimeter and projection surface ar-ea obtained by image analysis were used to calculate Sphericity Coefficient (SC) by the following equation [35].

(1) $$SC=\frac{4\pi A}{P^{2}},$$

where A is the projected bead surface area and P its perimeter.

### 2.4. Calorimetric Analysis

Betamethasone, polymers, blank and B loaded beads were analysed by differential scanning calorimetry (DSC) on an indium calibrated apparatus (DSC 822e, Mettler Toledo, Columbus, OH, USA). Thermograms were recorded on accurately weighed quantities of each sample in a 40 µL aluminium pan, sealed and pierced. The samples were heated from 25 to 350 °C at a scanning rate of 20 °C/min in nitrogen atmosphere at a flow rate of 150 mL/min. Melting temperature and peaks intensity were measured for all samples and compared with each other. The analyses were carried out in triplicate.

### 2.5. Drug Content and Encapsulation Efficiency

Samples of beads from each batch (about 10–15 mg) were dissolved under vigorous stirring in PBS buffer (100 mM, pH 6.8). Afterwards, 23 mL of ethanol were added and the suspension was centrifuged at 6000 rpm for 10 min. The Actual drug content (ADC) was obtained analysing the solution spectrophotometrically at 244 nm using an Evolution 201 UV/VIS Spectrometer, Thermo Scientific (Waltham, MA, USA). The analytic method was validated for linearity in the range of 3.3–20 μg/mL (*R*^2^ = 0.9999 and RSD < 2%). Encapsulation efficiency (EE) was calculated as the percentage ratio of actual to theoretical drug content (TDC) i.e., the weight of drug added (g)/weight of polymers and drug added (g) × 100. Each analysis was performed in triplicate; results were expressed in terms of mean ± standard deviation.

### 2.6. Drug Release Studies

The USP dissolution Apparatus II (AT7 Smart, Sotax, Allschwil, Switzerland) was used to obtain information on B release from the produced beads, at the following experimental conditions: paddle, 75 rpm, 37 °C on line with a UV spectrophotometer (Lambda 25 UV/VIS Spectrometer, Perkin Elmer, Waltham, MA, USA). A classic pH-change assay, method A, accordingly to ˂1092˃ monograph “The Dissolution Procedure: Development and Validation” (USP 36), was used. So, an accurately weighed amount of beads containing about 10–15 mg of betamethasone, in order to meet the sink conditions, was plunged into 750 mL 0.1M HCl for 2 h (acid stage); then 250 mL of 0.2 M Na_3_PO_4_ were added and pH was adjusted to 6.8 (buffer stage). Dissolution tests were conducted on six different batches of particles; mean values and standard deviation were evaluated.

### 2.7. Swelling Degree Analysis

Swelling behaviour of the beads was evaluated in simulated gastric fluid (SGF) for 120 min and successively in simulated intestinal fluid (SIF) until complete erosion was observed. At pre-determined times, beads were withdrawn from the dissolution vessel, dabbed with paper to remove the water on the bead surface and then weighted. The swelling ratio (SwR) for each sample was calculated according to Equation (2) [48]:

(2) $$Swelling\;Ratio=\frac{bead\;weight\;at\;time\;t-bead\;weight\;at\;time\;zero}{bead\;weight\;at\;time\;zero}$$

## 3. Results and Discussion

### 3.1. Core-shell Beads’ Production and Characterization 

Core-shell beads consisting of zinc pectinate and betamethasone (B) in the core and zinc alginate in the shell were manufactured by prilling technology in co-axial configuration. This system is based on the use of two concentric nozzles, one for the inner core and the other one for the annular solution. During the process, the polymeric jet is broken-up by radiofrequency into uniform core-shell droplets that drip into a Zn^2+^ gelling solution and “solidify”. Polymeric feed solutions’ composition were set according to our previous research experiences [40]. In detail, the main formulations (F3-F4-F5) were designed fixing pectin core composition (polymer concentration 4.0% *w/v*; B/pct mass ratio 1:20) and modifying alginate solution concentration from 1.0% to 2.0% *w/v*, that is, decreasing core/shell mass ratio from 4:1 to 2:1 (Table 1). As control, “only core” particles of alginate (F1) and pectin (F2) were also produced using prilling apparatus in its standard configuration (mono nozzle). Gelling conditions were set as follow: Zn^2+^ concentration = 10% *w/v*, pH = 1.5, cross-linking time = 8 min, to reach a compromise between the mechanical strength of the beads and the drug loss into the gelling solution.

Good encapsulation efficiency (E.E.) values were obtained for all dried beads (E.E. ranging from 71.6% to 84.8%, Table 1). As expected, higher values were recorded for core-shell particles. In this case, the presence of the shell reduces the betamethasone leak from the core to the gelling medium during the polymer crosslinking phase; accordingly, a better entrapping of the drug into the pectin core was achieved. Particularly, the specific core-shell mass ratio used to formulate the particles affects encapsulation efficiency values; the lower the core-shell mass ratio, the higher the EE. In fact, B encapsulation was higher for F4 (core-shell mass ratio 2.7:1) and F5 (core-shell mass ratio 2:1) with values up to 85%. The decrease in the core-shell mass ratio corresponds to an increase in the concentration of alginate. Therefore, the cohesive forces inside the shell increase too; this determines an enhancement of shell matrix texture very useful to reduce drug migration from the inner core during the crosslinking step. 

Dimensional distribution analyses displayed diameters around 2 mm for “only core” formulations (F1 and F2) and in the range of 2.5–2.9 mm for core-shell beads (F3, F4, F5) (Table 1). So, even if the external nominal diameter of the extrusion system is the same (i.e., 600 µm for both mono and co-axial apparatus), the particle size is considerably different. In particular, for core-shell formulations, the lower the core-shell mass ratio, the higher the mean diameter; in this case, the increase in the alginate concentration determines a high entanglement between the polymeric chains that makes the polymer jet highly cohesive. This effect delays drops detachment from the co-axial nozzle edge leading to an increase in size of any single droplets coming out from the nozzle before vibration was able to detach them from it. 

Beads shape was evaluated by means of the sphericity coefficient (SC). Regarding “only core” particles, very high sphericity (0.93) was obtained for alginate beads (F1) whereas lower (0.86) for pectin ones (F2). Pectin-alginate beads showed SC data similar to pectin “only core” particles, with values ranging between 0.83 and 0.88. 

**Figure 1:**
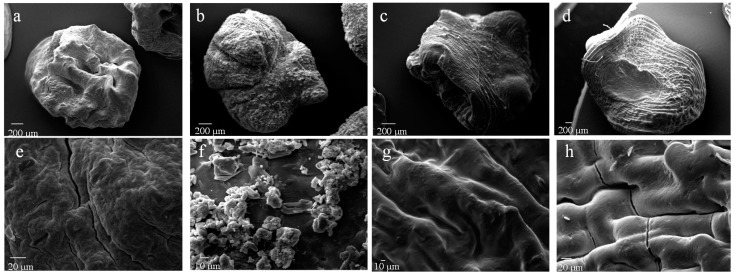
SEM microphotographs at different magnification of formulations: F1 (**a**,**e**), F2 (**b**,**f**), F3 (**c**,**g**) and F5 (**d**,**h**).

**Figure 2:**
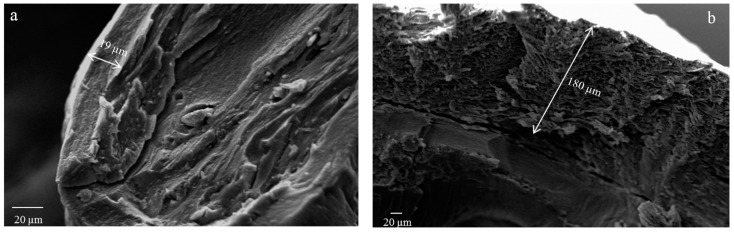
SEM microphotographs of cryofractured core/shell beads: F4 (**a**) and F5 (**b**).

SEM investigation highlighted significant differences in terms of particle surface and inner structural properties. As shown in Figure 1, F1 (alginate beads) showed an almost spherical but highly corrugated surface whereas F2 (pectin beads) appeared less regular in shape and with a reduced surface roughness. Formulations F3, F4 and F5 (pectin-alginate beads) showed a low sphericity and retained the corrugated appearing typical of the alginate that forms the outer shell. This aspect can be correlated to the effectiveness of the coating phase highlighting that in the selected formulation and process conditions, the pectin core is entirely covered by a homogeneous alginate shell. In fact, as evidenced by cross-section micrographs (Figure 2), there is a distinct interface between the core and the shell. In addition, the higher the shell polymeric concentration, the higher the thickness.

SEM analysis at higher magnification showed that pectin beads (F2) exhibited on the surface numerous spots of betamethasone crystals. This phenomenon may be due to the poor ability of zinc pectinate gel to effectively retain into its texture the loaded drug that, during the drying process partly migrates on the surface. On the contrary, analysing pectin-alginate particles, the presence of drug crystals was highly reduced. In fact, as shown in Figure 1g,h only tiny spots of the drug on beads surface were detected.

### 3.2. Calorimetric Analysis

**Figure 3:**
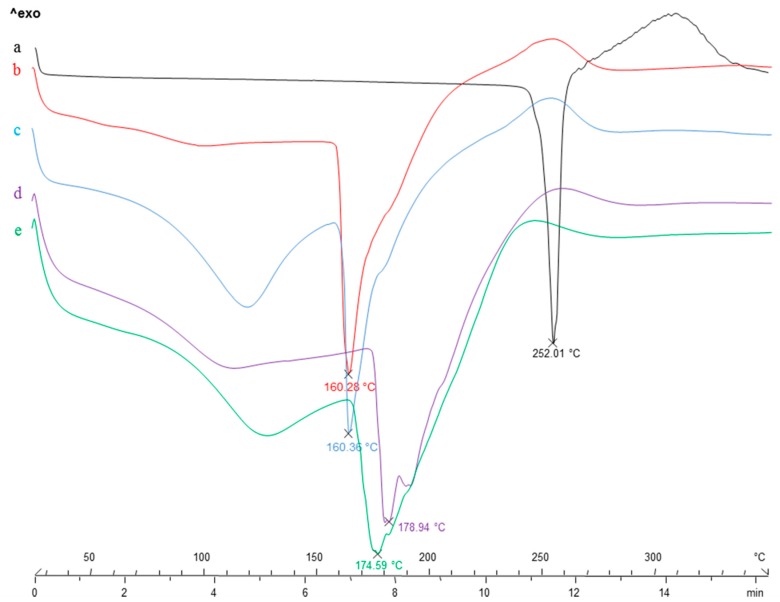
DSC thermal profiles of: B raw material (**a**), “only core” beads of alginate, both blank F1_b (**b**) and B loaded F1 (**c**) and “only core” beads of pectin, both blank F2_b (**d**) and B loaded F2 (**e**).

With the aim to highlight potential drug-polymeric core interactions as well as to evaluate the solid state of the drug after prilling process, DSC experiments were performed [49]. DSC thermal profiles of B raw material, blank and B loaded “only core” beads of alginate (F1_b and F1) and pectin (F2_b and F2) are shown in Figure 3. 

Thermal profile of crystalline betamethasone exhibited a very narrow melting peak at 256 °C and a broad exothermic peak around 310 °C due to the oxidative degradation (Figure 3a). The thermogram of blank alginate beads F1_b (Figure 3b) showed an endothermic event around 160 °C corresponding to the melting of zinc-alginate matrix, followed by a ramp-like event ranging from 220 to 280 °C referable to cross-linked matrix degradation. B loaded alginate beads F1 (Figure 3c) showed a trend comparable to blank beads (F1_b), but characterized by an additional broad endothermic signal in the thermal range 100–130 °C corresponding to the loss of hydration water from the polymeric matrix. It is probably that the presence of the drug inside the egg-box increases its water absorption ability. Regarding pectin-based formulations, blank beads F2_b (Figure 3d) showed an endothermic event around 180 °C due to the melting of the zinc pectinate matrix and an exothermic signal ranging from 240–260 °C due to its degradation. A similar trend was recorded for B loaded pectin beads F2; in this case the dehydration band was larger and probably responsible of the shift at lower temperatures (around 175 °C) of the main endothermic event due to zinc pectinate matrix melting. 

The DSC thermograms of the analysed drug-loaded beads did not give any evidence of betamethasone melting, confirming the entrapment in the polymer matrix as a consequence of its physical interaction with the core polymer. 

FT–IR studies were conducted to confirm interactions between betamethasone and the polymer matrix in the loaded particles and the results are reported as Supplementary Materials. 

### 3.3. Study of Drug Release and Swelling Degree

**Figure 4:**
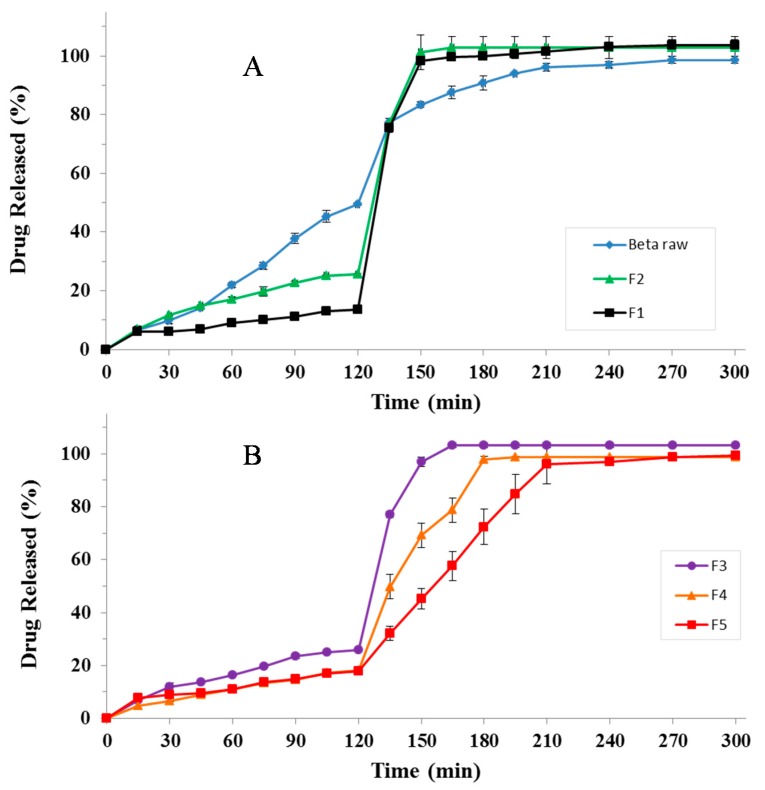
Release profiles of B raw material, formulations F1-F2 (**A**), F3, F4 and F5 (**B**). Mean ± SD (*n* = 6).

To verify the ability of the produced particles to modulate betamethasone release in the gastrointestinal tract, dissolution tests were performed by means of the USP Apparatus II [50], using a classic pH change protocol. The dissolution profiles are shown in Figure 4.

As shown in Figure 4, all polymeric particles were able to modulate drug release in simulated gastro-intestinal fluids compared to pure betamethasone; in this case around 50% of the total amount of commercially available pure drug dissolved in SGF whereas almost total dissolution was achieved after about 210–240 min (Figure 4A). Particularly, “only core” formulations (F1 and F2) were able to significantly reduce the total amount of drug released in SGF reaching the range 10–25% (~10% for alginate beads F1; ~25% for pectin beads F2) and allowing the complete drug release in SIF, after pH change, in about 30 min (experiment time point: 150 min). In fact, as reported elsewhere the phosphate ions in SIF determine the capture of zinc ions from the cross-linked polymeric matrix, causing its dissolution with consequent erosion and release of the encapsulated drug [51]. The only core pectin beads F2 showing the presence of numerous untrapped B crystals on particle surface (Figure 1b,f) rapidly getting in contact with the release medium give a high release rate in gastric fluid. Betamethasone release profiles from core-shell systems which showed only tiny spots of the drug on beads surface (as shown in Figure 1c,g for F3 and in Figure 1d,h for F5) resulted similar during the acidic cycle but significantly different in the successive step (Figure 4B). Particularly, in SGF formulation F3 showed a drug release of about 25%, F4 and F5 delivered less than 20% of B. Similarly, in SIF, the drug release was slower for F4 and F5, compared to F3, due to the alginate shell that is able to delay beads hydration/dissolution/erosion processes and, consequently, drug release in the simulated gastro-intestinal fluids. The higher the shell thickness, the slower the dissolution/release phenomena. In fact, among core-shell systems, F5, characterized by a greater alginate thickness, exhibited the lowest drug release rate due to a more difficult access of the fluid to the inner core of the beads. 

**Figure 5:**
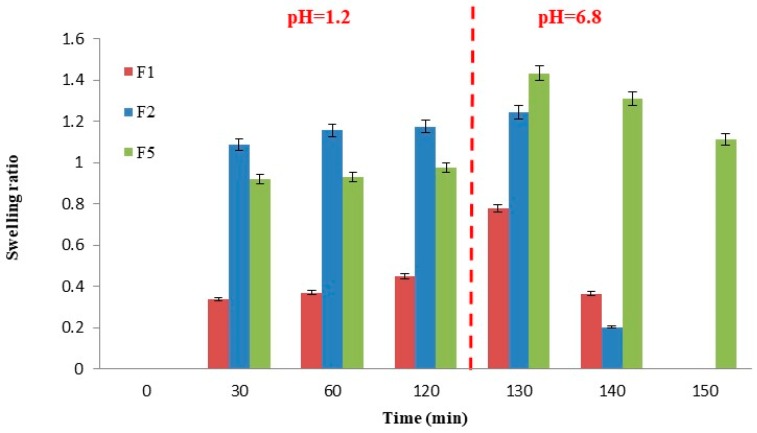
Swelling profiles of formulation F1 and F2 compared to F5. Mean ± S.D.; (*n* = 3).

In order to verify the influence of beads’ swelling ability on their release properties, swelling experiments were performed. The main results are reported in Figure 5. 

Alginate “only core” formulations (F1) exhibited low swelling properties in SGF with mean SwR values around 0.45 and retained the same volume as long as pH remained constant at 1.0; after pH change, at the time point of 130 min, particles reached their maximum swelling ratio of 0.78; after that beads structure starts to disaggregate due to the mechanism of capturing of the zinc ions by phosphate ions contained in SIF buffer and complete drug release occurs (100% of drug release after 150 min). Pectin “only core” formulations (F2) showed higher SwR both in SGF and SIF with values of 1.09, already at *t* = 30 min, and 1.24 at *t* = 130 min. Swollen beads rapidly disaggregated, explaining betamethasone fast release in SIF observed during the dissolution tests. Core-shell particles of formulation F5 showed a halfway swelling behaviour between F1 and F2 in SGF but a higher swelling ratio (1.43) after 130 min in SIF. In this case, the swollen matrix of the outer layer acts as a barrier delaying the hydration of the inner polymeric core and thus, the dissolution/erosion phenomena determining a sustained drug release in simulated intestinal fluid (100% of drug release after 240 min).

## 4. Conclusions 

This study confirms the great versatility of prilling technique for the production of controlled DDS, highlighting the importance of both microencapsulation system configuration and polymeric excipients choice. These aspects, if properly set, can determine the development of uniform core-shell particles able to modulate, after oral administration, the release of drugs with different chemical-physical and biopharmaceutical characteristics. Particularly, the optimized core-shell formulation F5 gives the possibility to prevent the early release of the drug in the upper part of the GIT thanks to the presence of the zinc-alginate gastro-resistant outer layer and to specifically deliver it in the colon thanks to the selectivity of zinc-ALM pectinate for this district. Therefore, these particles could be useful for colonic selective delivery of betamethasone as required for IBD treatment.

## Figures and Tables

**Table 1:** Formulation code, polymer, feed solutions’ composition, Actual Drug Content (ADC), Encapsulation Efficiency (EE), mean diameter and sphericity coefficient (SC) of only-core beads obtained by mono-nozzle prilling (F1 and F2) and core-shell beads manufactured by co-axial prilling (F3-F4-F5).

Formulation Code	Polymer (% *w/v*)	Core:Shell Mass Ratio	Drug:Core Mass Ratio	ADC (% ± SD)	EE (% ± SD)	Mean Diameter (mm ± SD)	SC
F1	Core: alg 2.0	/	1:20	3.4 ± 0.1	71.6 ± 2.6	2.01 ± 0.12	0.93 ± 0.01
F2	Core: pct 4.0	/		3.5 ± 0.1	72.8 ± 2.2	1.94±0.11	0.86 ± 0.04
F3	Core: pct 4.0 Shell: alg 1.0	4:1		2.9 ± 0.2	75.0 ± 4.2	2.50 ± 0.15	0.83 ± 0.03
F4	Core: pct 4.0 Shell: alg1.5	2.7:1		2.9 ± 0.1	81.1 ± 3.0	2.72 ± 0.11	0.86 ± 0.04
F5	Core: pct 4.0 Shell: alg 2.0	2:1		2.7 ± 0.1	84.8 ± 3.7	2.93 ± 0.31	0.88 ± 0.04

## Supplementary Materials

The following are available online at https://www.mdpi.com/1999-4923/12/2/87/s1, Figure S1: FT–IR spectra of: B raw material, blank F3 and B loaded F3.
