# Supplementary material for: Pectin and Zinc Alginate: The Right Inner/Outer Polymer Combination for Core-Shell Drug Delivery Systems
Source: Pharmaceutics. 2020 Jan 21;12(2):87. doi: 10.3390/pharmaceutics12020087 (PMC7076462; doi:10.3390/pharmaceutics12020087)
Supplement: Supplementary file 1 [file pharmaceutics-12-00087-s001.pdf]

pharmaceutics

Pectin and Zinc Alginate: The Right Inner/Outer Polymer Combination for Core-Shell Drug Delivery Systems

Giulia Auriemma, Andrea Cerciello, Rita P. Aquino, Pasquale Del Gaudio, Bruno M. Fusco and Paola Russo

FT–IR studies were conducted in order to verify the presence of interactions between betamethasone and the polymer matrix in the loaded particles. Infrared analysis was performed using a FTIR spectrophotometer (IRAffinity-1S, Shimadzu Corporation, Kyoto, Japan) equipped with a MIRacle ATR accessory with ZnSe crystal plate. The samples were directly charged over the crystal plate and analysed using 256 scans with a 1 cm^{-1} resolution step. Each experiment was carried out in triplicate, and results averaged.

As shown in Figure S1, betamethasone presented the asymmetric stretching bands of the carboxylate anion between 1710 and 1600 cm^{-1} and the symmetric ones at 1430 cm^{-1}; blank pectin particles presented amide bands between 1680 cm^{-1} and 1592 cm^{-1} while COO stretching was observed at 1410 cm^{-1}. Drug loaded particles show the betamethasone asymmetric COO stretching bands shifted at lower wavenumbers (between 1650 and 1600 cm^{-1}), while symmetric COO stretching probably was covered by polymer matrix bands due to interactions with pectinate amide groups via Zn^{+2} coordination.

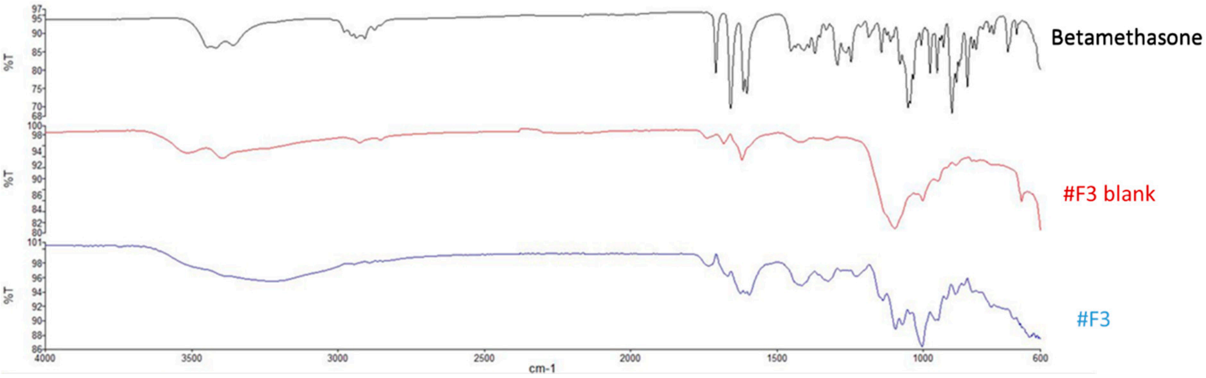

Figure S1. FT–IR spectra of: B raw material, blank F3 and B loaded F3.
